# Comparison of urine and semen samples for HPV detection: a cross-sectional study in fertility clinic patients

**DOI:** 10.1128/jcm.00882-25

**Published:** 2025-10-30

**Authors:** Dayanara Delgado López, Andrea A. Cabrera-Andrade, Roque Rivas-Párraga, Pedro José Gonzalez, Bernardo Vega Crespo, Vivian Alejandra Neira

**Affiliations:** 1 Faculty of Medical Sciences, University of Cuenca 27889, Cuenca, Ecuador; 2 Faculty of Medicine and Health Sciences, University of Antwerp 26660, Antwerp, Belgium; 3 Faculty of Chemical Sciences, University of Cuenca 27889, Cuenca, Ecuador; 4 Biomass to Resources Group, Universidad Regional Amazónica Ikiam, Napo, Ecuador; 5 Faculty of Medicine, University of Azuay 27892, Cuenca, Ecuador; 6 Fertility Unit, Hospital del Río, Cuenca, Ecuador; 7 Department of Biosciences, Faculty of Chemical Sciences, University of Cuenca 27889, Cuenca, Ecuador; Wadsworth Center - NYSDOH, Albany, New York, USA

**Keywords:** male self-sampling, non-invasive male screening, HPV genotyping

## Abstract

Human papillomavirus (HPV) is a prevalent sexually transmitted infection linked to genital warts and various cancers. While effective screening methods exist for women, detecting HPV in men remains challenging due to asymptomatic infections and the lack of standardized, non-invasive diagnostic tools. This study aimed to assess the correlation between HPV DNA detection in urine and semen samples from men attending a fertility clinic in Ecuador. This cross-sectional study included 106 sexually active male patients referred for semen analysis at the Fertility Unit of Hospital del Río, Cuenca, Ecuador, between June 2024 and March 2025. Each participant provided paired semen and urine samples, which were analyzed for HPV genotyping. Sociodemographic and behavioral data were also collected. HPV DNA was detected in 15% of semen samples and 9.4% of urine samples. Both low- and high-risk genotypes were identified in semen and urine. In semen samples, high-risk HPV was detected in 9.4% and low-risk HPV in 5.7%. In urine, high-risk HPV was detected in 8.5% and low-risk HPV in 0.9% of samples. Among high-risk genotypes, HPV 58 was the most frequent in urine (20%), while HPV 81 was the most frequent in semen (19%). Genotypes such as HPV 16, 18, 39, 52, and 66 were detected exclusively in semen. Compared with semen sampling, urine sampling showed a sensitivity of 37.5% and a specificity of 95%. Agreement between the two sample types was fair (kappa = 0.39). Although urine sampling offers a non-invasive option, its limited sensitivity restricts its utility as an HPV detection method in asymptomatic men. Semen analysis provides higher detection rates and may be more reliable for HPV screening in male fertility populations.

**IMPORTANCE:**

This study addresses a critical knowledge gap regarding human papillomavirus (HPV) detection in men, a topic insufficiently covered in current control and prevention strategies. While screening and vaccination efforts in women have advanced considerably, the absence of standardized, well-accepted, and non-invasive methods for HPV detection in men limits the recognition of their roles as reservoirs and transmitters in the HPV transmission pathway. Evaluating alternative solutions for HPV testing, such as urine and semen sampling, not only contributes valuable scientific knowledge to improve detection in this population but also establishes the foundation for public health policies on male HPV testing. Ultimately, this work supports the goal of reducing global HPV prevalence and its associated diseases.

## INTRODUCTION

Human papillomavirus (HPV) is one of the most common sexually transmitted infections worldwide, associated with a spectrum of anogenital and oropharyngeal diseases, including genital warts and several cancers (1, 2). While HPV screening and prevention strategies have been well developed for women, particularly through cervical cytology and HPV DNA testing, HPV detection in men remains less standardized. Male HPV infections often go undiagnosed due to the asymptomatic nature of the infection and the absence of a universally accepted, non-invasive screening method (3, 4).

In line with current recommendations, HPV vaccination is primarily offered to girls according to the WHO (5). However, female-only vaccination is insufficient to eradicate HPV infection or to achieve the 90–70–90 target (6, 7). According to Garolla et al., women have more than 80% probability of acquiring HPV infection, while men have a risk exceeding 90% (8). Currently, three vaccines approved by the US Food and Drug Administration (FDA) are available, all of which cover HPV types 16 and 18, among others (9). Vaccination of boys should be strongly encouraged, as the ultimate goal of HPV immunization is the eradication of cervical cancer and other HPV-related diseases through a gender-neutral vaccination strategy (10). In Ecuador, the Ministry of Public Health (MSP) has traditionally vaccinated only girls aged 9 to 11 years; however, since May 2024, vaccination has been extended to both sexes (11, 12).

Traditional methods for HPV detection in men rely on clinician-collected swabs from the penile shaft, urethra, or anal region. However, these approaches are invasive, may cause discomfort, and have variable sensitivity depending on the anatomical site and sampling technique (13). Importantly, no universally validated or widely accepted method for HPV detection in the male population currently exists (14). In response to these limitations, urine sampling has emerged as a non-invasive, self-collectable alternative. Several studies have demonstrated that urine samples, particularly first-void urine, can detect HPV DNA in males with moderate sensitivity (15–17). For example, Golijow et al. detected high-risk HPV in 18.7% of urine samples from men in a high-risk population in Argentina, and Jin et al. found a prevalence of 35.1% among asymptomatic male partners of women with sexually transmitted infections (17, 18).

Nonetheless, the clinical performance of urine-based HPV testing in men remains suboptimal. Aung et al. found that urine sample positivity varied significantly by circumcision status and the anatomical location of genital warts, with lower detection in circumcised men and those with penile shaft lesions (19). Similarly, a study performed by Koene et al. compared urine and penile swabs and reported higher detection in swabs (61.4%) than in urine (35.1%) (20). These findings are echoed in the review by Enerly et al. who emphasized the need for standardized protocols and noted that variability in sample processing and assay choice significantly affects detection rates (21).

Although urine has been widely studied, semen remains an underexplored but biologically plausible sample type for HPV detection in men. HPV DNA has been detected in semen in multiple studies, and a recent systematic review and meta-analysis by Laprise et al. reported prevalence rates ranging from 2% to 31% across populations and methodologies (22). The presence of HPV in semen has also been associated with impaired sperm quality, suggesting a potential role in fertility and viral shedding (23–25). Despite these implications, semen is rarely used in routine HPV screening or research protocols.

A small number of studies have begun to address this gap. Gupta et al. (26) included semen among several sample types for HPV genotyping and noted its potential utility in improving the accuracy of male risk profiling, while Melchers et al. demonstrated that HPV DNA could be successfully detected in semen using PCR techniques (16, 26).

Given the limitations of urine sampling and the biological relevance of semen, there is a clear need to compare these two non-invasive sample types for their performance in HPV detection among men. The primary objective of this study was to evaluate the correlation between urine and semen samples for the detection of HPV DNA in male patients attending a fertility clinic. The aim was to determine whether urine could serve as a reliable, non-invasive alternative to semen for HPV genotyping in this population. To the best of our knowledge, this is the first study of its kind conducted in Ecuador.

## MATERIALS AND METHODS

### Study design and setting

This was an observational, cross-sectional, and analytical study conducted at the Fertility Unit of Hospital del Río in Cuenca, Ecuador. The study period spanned from June 2024 to March 2025. The sociodemographic characteristics and sexual history of the population can be observed in Table 1.

**TABLE 1:** Sociodemographic characteristics and sexual history of the study population, *n* = 106

Variable	*N* (%)
Age: Mean = 36.83; Mode 34; SD ± 6.96	
21 to 29	10 (9.4)
30 to 39	65 (61.3)
40 to 49	25 (23.6)
50 to 59	5 (4.7)
60 to 62	1 (0.9)
Educational level	
Primary school	3 (2.8)
High school	25 (23.6)
University	78 (73.6)
Marital status	
Married	71 (68.3)
Divorced	4 (3.8)
Single	21 (20.2)
In a consensual union	8 (7.7)
Has biological children	
Yes	25 (23.6)
No	81 (76.4)
History of abortion	
Yes	24 (22.9)
No	81 (77.1)
Number of abortions: mean = 0.36; mode 0; SD ± 0.56	
0	51 (68)
1 to 2	24 (32)
Age at first sexual intercourse: mean = 17.44; mode 17; SD ± 2.93	
12 to 14	11 (10.4)
15 to 19	77 (72.6)
20 to 24	16 (15.1)
25 to 29	1 (0.9)
30 to 34	1 (0.9)
Previous HPV Diagnosis	
Yes	4 (3.8)
No	102 (96.2)
Number of lifetime sexual partners: mean 8.32; mode 5; SD ± 9.5	
1 to 4	36 (34.0)
5 to 9	40 (37.7)
10 to 29	25 (23.6)
30 to 49	4 (3.8)
50 to 75	1 (0.9)
Use of protection during sexual intercourse	
Yes	66 (62.3)
No	40 (37.7)
Sexual activity with high-risk partners	
Yes	24 (22.6)
No	82 (77.4)
Has undergone HPV testing	
Yes	8 (7.5)
No	98 (92.5)
Days of ejaculatory abstinence: mean = 3.19; mode = 3; SD ± 0.82	
2 to 4	70 (68.0)
4 to 6	32 (31.1)
6 to 7	1 (1.0)
Reason for semen analysis	
Fertility	99 (93.4)
Other (comparison with sibling, pelvic pain, low testosterone, other)	7 (6.6)

### Study population

Eligible participants were male patients referred to the Fertility Unit for semen analysis. All individuals were invited to participate by the attending fertility specialist. To be included in the study, participants had to meet the following criteria: be ≥18 years of age, be sexually active, have abstained from sexual activity for at least 72 h prior to sample collection, and have been referred for semen analysis (semen parameters are presented in Table 2 according to HPV status). Patients diagnosed with varicocele or with a history of vasectomy were excluded. A total of 106 patients were included, each of whom provided both a semen and a urine sample.

**TABLE 2:** Seminal parameters in the study population according to HPV presence

Variable	Global *n*	HPV- *n*	HPV+ *n*
Number of individuals	106 (100%)	90 (84.9%)	16 (15.1%)
Concentration ≤15 × 10⁶/mL, *n* (%)	11 (10.4%)	8 (8.9%)	3 (18.8%)
Progressive motility (%) Median (IQR)^*a*^ Range	45.5 (30-57.8) 3-80	48 (32-59.8) 3-80	34.5 (20.2-46) 6-65
Progressive motility ≤32%, *n* (%)	32 (30.2%)	24 (26.7%)	8 (50%)
Normal morphology (%) Median (IQR) Range	3 (2-4) 1-10	3 (2-4) 1-10	2.5 (2-3) 1-4
Normal morphology ≤4%, *n* (%)	89 (84%)	73 (81.1%)	16 (100%)
pH Median (IQR) Range	8.1 (8-8.3) 7-9	8.1 (7.9-8.3) 7-9	8.1 (8.0-8.3) 7-9
Normal viscosity, *n* (%)	88 (83%)	76 (84.4%)	12 (75%)

^*a*^ The IQR, or interquartile range, is a measure of statistical dispersion that indicates how spread out the central 50% of a data set is. It is calculated as the difference between the third quartile (Q3) and the first quartile (Q1). Essentially, it shows the range within which the central portion of the data lies.

### Sample collection

Following informed consent, participants completed a standardized data collection form that included sociodemographic information and details regarding their sexual and reproductive history. Each participant received two sterile containers, one for urine and one for semen collection. Participants were instructed to first collect the urine sample without prior cleansing of the genital area, capturing the first-void urine until a volume of 50 mL was reached. Subsequently, a semen sample was obtained via masturbation.

All specimens were anonymized using an alphanumeric code and promptly transported to the Molecular Biology Laboratory of the University of Cuenca. Urine samples were centrifuged within a maximum of 6 h post-collection to prevent degradation of biological material.

### HPV genotyping

Genomic DNA was extracted from both urine and semen samples for HPV genotyping. Urine samples were centrifuged at 3,000 rpm for 15 min to obtain a cellular pellet used for DNA extraction. For semen samples, 300 µL of ejaculate was processed. DNA extraction was performed using the Invitrogen commercial kit (Jetflex Genomic DNA Purification kit), following the manufacturer’s protocol for both types of specimens.

HPV genotyping was performed using the 24 HPV Typing Kit (Jiangsu Mole Bioscience), which enables the identification of HPV genotypes through real-time PCR. The identified genotypes were categorized as follows: low-risk types are (6, 11, 42, 43, 44, and 81), and high-risk types (16, 18, 26, 31, 33, 35, 39, 45, 51, 52, 53, 56, 58, 59, 66, 68, 73, and 82). All samples were processed with the inclusion of an internal amplification control, as well as positive and negative controls. Amplification was carried out using the Exicycler 96 thermal cycler (Bioneer). The amplification protocol followed the manufacturer’s recommendations.

### Data analysis

All data from the data collection form were entered into an Excel spreadsheet for analysis. Descriptive statistics were used to characterize the study population, employing the R software package for statistical analysis. To assess the level of agreement between urine and semen samples for HPV detection, the kappa statistic was calculated. A kappa value of 0 indicated no agreement better than chance, while a value of 1 represented perfect agreement. Intermediate kappa values were interpreted as follows: 0.00–0.20 (poor agreement), 0.21–0.40 (fair agreement), 0.41–0.60 (moderate agreement), 0.61–0.80 (good agreement), and >0.81 (excellent agreement).

Additionally, the sensitivity, specificity, positive predictive value (PPV), and negative predictive value (NPV) of urine analysis were calculated, using semen analysis as the reference method. These measures were computed to evaluate the diagnostic accuracy of urine samples for HPV detection in comparison to semen samples.

## RESULTS

The mean age of the participants was 36 years, with 61% between 30 and 39 years old. Regarding educational level, 73% had attended university. Additionally, 68% were married, and 76% had not had biological children. A total of 77% reported no history of abortion, and 72% had their first sexual intercourse between the ages of 15 and 19 years. Furthermore, 96% had no prior HPV diagnosis, and 37% reported having had 5 to 9 sexual partners. A total of 62% stated that they used protection during sexual intercourse, and 77% reported not having sexual activity with high-risk partners. Moreover, 92% had never undergone HPV testing. Regarding ejaculatory abstinence, 68% of men reported 2 to 4 days. Finally, 93% indicated that the reason for semen analysis was fertility assessment.

In Table 2, seminal parameters are presented. Overall, 15.1% of individuals were HPV positive. In this group, progressive motility was lower (34.5 [20.2–46]) compared with the HPV-negative group. Most participants exhibited sperm morphology values at or below the WHO lower reference limit (≤4%), with this alteration observed in 84% of the total study population, 81.1% of HPV-negative individuals, and 100% of HPV-positive individuals. No differences in pH were observed between the groups.

The overall prevalence of HPV in semen samples was 15.1%, compared with 9.4% in urine samples (Table 3). Specifically, high-risk HPV was detected in 9.4% of semen samples and 8.5% of urine samples. Low-risk HPV was identified in 5.7% of semen samples and 0.9% of urine samples. Notably, urine samples also demonstrated the capacity to detect high-risk genotypes.

**TABLE 3:** Overall prevalence and prevalence of HPV in urine and semen samples, *n* = 106

Sample	Global *n* (%)	High-risk HPV^*a*^ *n* (%)	Low-risk HPV *n* (%)
Semen	16 (15.1)	10 (9.4)	6 (5.7)
Urine	10 (9.4)	9 (8.5)	1 (0.9)

^*a*^ The high-risk category includes all cases in which coinfection with low-risk HPV genotypes was also detected (2 [1.9%] and 4 [3.8%] for semen and urine, respectively).

The distribution of HPV genotypes between semen and urine samples revealed notable differences in detection patterns (Table 4). Several high-risk genotypes—such as HPV types 16, 18, 39, 52, and 66—were exclusively observed in semen samples. HPV types 6, 31, 42, 44, 45, 53, 56, 58, 59, and 81 were detected in both semen and urine samples. Among the high-risk genotypes, HPV type 58 was the most frequently detected in urine samples (20%) compared with semen samples (9.5%). Regarding low-risk HPV, type 81 was the most frequently detected in semen samples (19%), while type 44 was the most common in urine samples (13.3%). Semen samples were considered the gold standard, as they demonstrated a higher overall detection rate. Although both semen and urine samples detected high-risk genotypes, urine samples failed to detect certain low-risk genotypes, which were identified exclusively in semen samples.

**TABLE 4:** Distribution of any type of HPV according to sampling method

Genotype	6^*b*^	16^*a*^	18^*a*^	31^*a*^	39^*a*^	42^*b*^	44^*b*^	45^*a*^	52^*a*^	53^*a*^	56^*a*^	58^*a*^	59^*a*^	66^*a*^	81^*b*^
Semen *n* (%)	1 (4.8)	1 (4.8)	1 (4.8)	1 (4.8)	1 (4.8)	2 (9.5)	2 (9.5)	1 (4.8)	1 (4.8)	1 (4.8)	1 (4.8)	2 (9.5)	1 (4.8)	1 (4.8)	4 (19.0)
Urine *n* (%)	1 (6.7)	0	0	1 (6.7)	0	1 (6.7)	2 (13.3)	1 (6.7)	0	1 (6.7)	2 (13.3)	3 (20.0)	2 (13.3)	0	1 (6.7)

^*a*^ High-risk HPV.

^*b*^ Low-risk HPV.

The correlation between urine and semen sampling for HPV detection showed that urine sampling had a sensitivity of 37.5%, indicating that only one-third of the positive cases detected by semen were also detected by urine. In contrast, the specificity was 95%, suggesting that urine sampling accurately identified the majority of HPV-negative cases. The PPV was 60%, meaning that 60% of the positive results were true positives. The NPV was 89.6%, indicating that approximately 90% of the negative results were true negatives. The kappa coefficient was 0.39, reflecting an equal agreement between semen and urine sampling. Although urine sampling demonstrated high specificity, its limited sensitivity suggests that it may not be a suitable alternative for HPV detection in the male population (Table 5).

**TABLE 5:** Sensitivity, specificity, predictive values, and kappa correlation between urine and semen samples

		Semen sampling		Sensitivity	Specificity	PPV	PNV	Kappa
		Positive *n* (%)	Negative *n* (%)	% (IC 95%)	% (IC 95%)	% (IC 95%)	% (IC 95%)	*n* (IC)
Urine sampling	Positive	6 (5.7)	4 (3.8)	37.5 (18.5–61.4)	95.6 (89.1–98.3)	60.0 (31.3–83.2)	89.6 (81.9–94.2)	0.39 (0.11–0.62)
	Negative	10 (9.4)	86 (81.1)					

## DISCUSSION

The present study evaluated the diagnostic performance of urine sampling in comparison to semen sampling for the detection of HPV in men. Regarding sociodemographic characteristics, more than 60% of participants were between 30 and 39 years of age, and the mean age at first sexual intercourse was 17 years, which is consistent with the findings of Malhotra et al. (27). Participants reported a mean of eight lifetime sexual partners, aligning with the data presented by Buttman et al. (28). Concerning protective sexual practices, 62.3% of participants reported consistent condom use during sexual activity, which is comparable to the findings of Borges et al. (29). However, this differs from Malhotra et al. (27), where over half of the study population reported not using protection. Additionally, 77.4% of participants in our study reported no history of sexual activity with high-risk partners, similar to Buttman et al. (28) and further supported by their later study (30).

Our findings show an overall HPV prevalence of 15.1% in semen samples compared with 9.4% in urine samples. These results are consistent with those reported by Lyu et al. (31), who found an overall prevalence of 11.4%. Lyu et al. (31) also reported a prevalence of high-risk HPV types in semen of approximately 10%, which is in line with the 8.5% prevalence observed in our study.

High-risk HPV types, including HPV 16, 18, 39, 52, and 66, were detected exclusively in semen samples and not in urine. This finding is in agreement with the study conducted by Astori et al. (32), which demonstrated a higher detection rate of HPV in semen compared to urine. This finding is particularly relevant, as HPV types 16 and 18 are responsible for more than 70% of precancerous cervical lesions and cervical cancers worldwide (33–36). These results suggest that semen sampling may represent an appropriate and minimally invasive diagnostic method for HPV detection in men.

Both low- and high-risk genotypes were identified in semen samples. The most frequent was HPV 81 (19%), followed by HPV types 42, 44, and 58, each with a prevalence of 9.5%. Clinically, the detection of high-risk types, such as HPV 16, 18, and 31, is highly relevant given their well-established association with intraepithelial lesions and anogenital cancers (9, 13, 14, 23, 37). These results highlight the primary role of men as transmitters and reservoirs in the HPV transmission pathway (18, 38). In addition, the detection of low-risk genotypes, typically associated with genital warts, underscores the importance of considering semen not only as a vector for viral transmission but also as a biological fluid in which a broad spectrum of HPV genotypes can be identified.

In terms of diagnostic accuracy, urine sampling showed high specificity (95.6%) but low sensitivity (37.5%), suggesting that it may not be an appropriate diagnostic tool for HPV detection in asymptomatic men. These values are compared with those reported by Koene et al. (20), who found a specificity of 95% and a sensitivity of 41%. Söderlund-Strand et al. (39) reported even lower sensitivity, at only 7.9% for urine samples. Although urine collection is convenient, non-invasive, and well-tolerated (17), its diagnostic utility in asymptomatic men is limited. Sensitivity improves in symptomatic individuals or when the sexual partner is infected (17).

While urine sampling remains appealing due to its feasibility and acceptability, its low sensitivity restricts its clinical applicability (20, 39). Conversely, although semen sampling may be perceived as slightly more invasive, it remains a well-tolerated, practical, and demonstrated a higher detection rate in our study, including identification of high-risk HPV types. These findings are consistent with Astori et al. (32), who also support semen as a viable diagnostic specimen for HPV detection.

In the context of fertility, previous studies have shown an association between HPV presence in semen and impaired sperm quality. Weinberg et al. (25), Moreno-Sepulveda and Rajmil (40), Foresta et al. (41), and Capra et al. (42) have reported a significant relationship between HPV infection and decreased sperm concentration, morphology, and motility. These findings suggest the need for further research to determine whether the higher prevalence of HPV observed in semen contributes to male infertility.

A limitation of this study is the absence of a general urine analysis, as samples were processed only by centrifugation to obtain cells for DNA extraction. This may have affected HPV detection and potentially contributed to lower sensitivity.

### Conclusion

In summary, while urine sampling offers clear advantages in terms of acceptability, feasibility, and non-invasiveness, its limited sensitivity restricts its utility for HPV diagnosis in asymptomatic men. Urine sampling may be more appropriate in symptomatic individuals or when a partner is known to be HPV-positive. Semen sampling, on the other hand, demonstrated a higher detection rate, including high-risk HPV types, and should be considered a convenient, minimally invasive, rapid, and reliable method for accurate HPV diagnosis.
